# Advanced disease programming brings much needed attention and improvements to inpatient paediatric HIV care in Mozambique

**DOI:** 10.1002/jia2.26203

**Published:** 2024-01-09

**Authors:** W. Chris Buck, Andreas Schindele, Elsa Taibo, Patricia Perez, Maria Inês Jorge Tomo de Deus, Mércia Matsinhe, Jessica Cowan, Teresa Beatriz Simione, Aleny Couto

**Affiliations:** ^1^ University of California Los Angeles, David Geffen School of Medicine Los Angeles California USA; ^2^ University of Witten/Herdecke Witten Germany; ^3^ I‐TECH Maputo Mozambique; ^4^ Centers for Disease Control and Prevention Maputo Mozambique; ^5^ United States Agency for International Development Maputo Mozambique; ^6^ Mozambique Ministry of Health Maputo Mozambique

1

In the early response to the HIV epidemic in Mozambique, paediatric antiretroviral treatment (ART) was principally available in day clinics, located in referral hospitals, with strong linkages between the inpatient wards and outpatient ART clinics. In 2013, the Ministry of Health (MoH) launched an acceleration plan that prioritized decentralization and scale‐up of ART services throughout the country [1]. The results of this effort have been remarkable with the comparison of key indicators from 2013 to 2021 demonstrating the percentage of health facilities offering ART increasing from 39% to 96%, the number of children on ART increasing from 41,400 to 99,169 and the estimated paediatric ART coverage increasing from 41% to 79% [2, 3].

To achieve these results, outpatient HIV care at primary health centres became the principal focus of programmatic attention, with an unintended negative impact on the quality of inpatient HIV care for children at referral hospitals. Programmatic data and local operational research demonstrated significant gaps at hospitals along the continuum of paediatric HIV care, including provider‐initiated testing and counselling (PITC), early infant diagnosis (EID) for HIV‐exposed infants, inpatient ART initiation and linkage to care post‐discharge [3, 4, 5, 6].

With the publication of guidelines for the management of advanced HIV disease (AHD) by the World Health Organization (WHO) in 2017, an opportunity to reengage with referral hospitals emerged with the recognition that despite massive scale‐up of prevention and treatment services nationally, there were still gaps in the quality of care for the sickest patients, many of whom require inpatient care. An activity for centrally led technical assistance for inpatient paediatric HIV care was launched in 2020 with direct support to the central or provincial referral hospital in each of the 11 provinces of Mozambique.

A standardized tool was developed by the MoH Pediatric HIV Technical Working Group to assess readiness to implement inpatient AHD services, including specific quantitative and qualitative indicators for areas that were already known to be problematic but were not well‐documented. The tool was then used by teams composed of provincial health HIV programme leadership, hospital staff and clinical partners (US government‐funded organizations that provide assistance with the delivery of clinical services to the Mozambique MoH) to conduct a baseline assessment at each hospital in the third quarter of 2020.

There were major diagnostic gaps noted with PITC coverage rates as low as 13% of audited charts despite MoH recommendations for universal opt‐out testing for inpatients [7]. The hospitals without paediatric inpatient clinical partner support had the lowest testing rates (59% mean compared to 93% in sites with partner‐supported counsellors). For inpatient EID, 4/11 hospitals had access to point‐of‐care testing, and only 2/7 hospitals without access reported a presumptive HIV diagnosis in the preceding 6 months. There was also wide variability in the capacity to trace infants whose DNA PCR results returned after discharge, with lower performance in hospitals without a supporting clinical partner.

Gaps in the quality of inpatient paediatric ART and tuberculosis (TB) care were also noted. Documentation of a nutritional evaluation for HIV‐exposed and positive children ranged from 0% to 100%, and only 5/11 hospitals had well‐functioning systems to ensure linkage to outpatient ART for patients newly diagnosed during hospitalization. Hospital pharmacy management was problematic with local medication stock‐outs during periods of adequate provincial supply including one hospital not having any paediatric ART formulations and 3/11 reporting recent interruptions in the availability of paediatric TB medications. And there were laboratory and diagnostic weaknesses noted, with only 4/11 hospitals performing CD4 testing, and limited capacity to perform sputum induction and gastric lavage to obtain specimens for TB diagnosis in younger children and infants.

With the formal launch of the Mozambique national AHD programme in 2021, activities were initiated to address gaps at each central and provincial hospital [8]. One of the principal causes identified for the gaps documented in the baseline readiness assessment was inadequate training of hospital staff, including physicians, nurses, pharmacists and laboratory staff, many of whom had never been included in a formal MoH HIV/ART or TB training and were unfamiliar with current guidelines. In addition, weaknesses in the supply chain management of key medications and laboratory tests, including CD4, were identified and prioritized for improvement.

Clinical partners made plans in coordination with provincial health directorates for intensified technical support to central and provincial hospitals to address the areas of weakness that were identified on the baseline assessment. Paediatric HIV trainings that included content on PITC/EID, ART, AHD and TB were performed for clinical and laboratory staff at each hospital. Routine supervision and technical assistance visits were made to each hospital, on‐call clinical support was provided for the discussion of cases and provincial working groups were established. Focus areas for these interventions included work to improve PITC and EID work flows, inpatient‐outpatient linkages, the availability and utilization of CD4 testing and site‐level procurement of paediatric HIV and TB medications. Targeted support was also delivered for the implementation of AHD screening using urine lipoarabinomannan (LAM) and cryptococcal antigen (CrAg) tests, which were newly available.

On the most recent round of AHD technical assistance and supervision visits to each hospital conducted in the second half of 2022 and the first half of 2023, modest improvements along the continuum of paediatric inpatient HIV care were noted (Table 1). For inpatient PITC, the mean percentage of admissions tested across all hospitals improved, and the site that had 13% coverage in the baseline assessment improved to 58%. Improved systems to monitor testing coverage had been implemented and some hospitals had new clinical partner‐hired counsellors. For inpatient EID, several hospitals without on‐site access to PoC testing had established referral pathways testing at nearby health centres with PoC machines or for prioritized processing of inpatient samples in nearby molecular biology laboratories. CD4 testing was available at all hospitals, and newly introduced urine LAM and CrAg testing were available at all sites, but with gaps in the use of these tests for eligible patients. And with the exception of one hospital which is newer and still has not yet established its paediatric HIV treatment programme, all other hospitals had in stock the paediatric formulations necessary to provide optimized dolutegravir‐based paediatric ART, cotrimoxazole preventive therapy and TB preventive treatment, and had active outpatient ART clinics for post‐discharge care of patients.

**Table 1:** Evolution of key selected indicators for inpatient paediatric HIV and TB care, aggregate results for central and provincial hospitals in Mozambique, 2020–2023

	Baseline assessment before AHD launch, 2020 (*n* = 11 hospitals)	Follow‐up assessment after AHD launch, 2022–2023 (*n* = 11 hospitals)
Dedicated HIV counsellors for inpatient paediatric PITC (#/% of hospitals)	5 (45%)	10 (91%)
% of sampled inpatient charts with documentation of PITC results (mean, range)	76% (13–100%)	94% (58–100%)
Access to timely EID testing (#/% of hospitals)	4 (36%)	9 (82%)
Availability of CD4 testing for hospitalized children (#/% of hospitals)	4 (36%)	11 (100%)
Both sputum induction and gastric lavage available for inpatient TB testing (#/% of hospitals)	4 (36%)	6 (54%)
% of sampled inpatient charts for HIV‐positive children with documentation of correctly dosed ART (mean, range)	42% (0–100%)	61% (13–100%)
Recent stock out of paediatric first‐line ART (#/% of hospitals)	5 (45%)	1 (9%)
Updated MoH paediatric HIV and TB job aids available on the ward (#/% of hospitals)	5 (45%)	10 (91%)

Abbreviations: ART, antiretroviral therapy; MoH, Ministry of Health; PITC, provider‐initiated HIV testing and counselling; PoC EID, point‐of‐care early infant diagnosis.

In conclusion, the implementation of the AHD programme has helped to rapidly identify and address gaps in inpatient paediatric HIV care at the provincial and central hospitals of Mozambique. The long‐term goal of the MoH is to prevent new paediatric HIV acquisitions, and when that fails, to diagnose and treat infants and children before they deteriorate to need inpatient care. But until the country is able to reduce its estimated 10% vertical transmission rate and move closer to its 95‐95‐95 paediatric goal (reported at 79%‐79%‐51% in 2021), the burden of HIV on paediatric inpatient wards will still be significant [3]. The renewed attention to paediatric inpatient care as part of AHD programming aligns well with recently updated advice from WHO on the provision of care to people with AHD who are seriously ill, and should continue to improve the quality of care at central and provincial hospitals, with likely similar benefits for rural, district and general hospitals as the national AHD programme expands [9].

## COMPETING INTERESTS

No competing interests to report.

## AUTHORS’ CONTRIBUTIONS

All authors supported activities through the Mozambique Pediatric HIV/ART Technical Working Group. WCB, AS, ET and PP led implementation activities. WCB drafted the manuscript. All authors contributed to the revision of the manuscript and approved the final version.

## FUNDING

This work has been supported by PEPFAR/CDC/HRSA: 6 NU2GGH002183‐01‐07 (University of California San Francisco, 2020–2021) and 5 U91HA068011400/UWSC12312 (I‐TECH/University of Washington, 2021–2022).

## DISCLAIMER

The findings and conclusions in this report are those of the authors and do not necessarily represent the official position of the funding agencies.

## Data Availability

The data that support the findings of this study are available from the corresponding author upon reasonable request.
